# Connected Surveillance for Detection of Complications After Early Discharge from Bariatric Surgery

**DOI:** 10.1007/s11695-020-04817-5

**Published:** 2020-07-21

**Authors:** Maud Neuberg, Marie-Cécile Blanchet, Benoit Gignoux, Vincent Frering

**Affiliations:** 1 Clinique de la Sauvegarde, Lyon, France; 2 Espace Médico-Chirurgical, Immeuble Trait d’Union, Entrée A29, Av des Sources, 69009 Lyon, France

**Keywords:** One-anastomosis gastric bypass, OAGB, Enhanced recovery after surgery, ERAS, bariatric surgery, Connected surveillance

## Abstract

As part of a bariatric enhanced recovery after surgery (ERAS) program, at-home follow-up using a novel Internet application was used to detect early complications. The study aimed to evaluate the safety and effectiveness of this “connected surveillance” protocol over a 10-day follow-up. Patients were monitored 24/7 by a trained nursing team with daily surgeon review of patient self-reports. Morbidly obese patients (*n* = 281) underwent OAGB (126, 47.70%) or sleeve gastrectomy (138, 52.3%). Of 264 who completed the study (mean age 40 years [20–66]), 3 (1.1%) underwent revision for early complications; there were 6 (2.1%) readmissions and 22 (8.3%) consultations. In a bariatric surgery ERAS program, “Internet-connected surveillance” proved safe and effective in detecting 100% of early complications, and most patients were satisfied with their care.

## Introduction

Enhanced recovery after surgery (ERAS) programs aim to improve multiple recovery parameters, reduce postoperative morbidity and length of hospital stay, and decrease costs. ERAS programs were pioneered nearly two decades ago, spearheaded in 2001 by the ERAS Working Group in Sweden [1]. ERAS protocols were studied initially in colorectal and orthopedic surgery [2, 3]; the field of bariatric surgery was also an early adopter of the concept [4]. One goal of ERAS programs is to reduce length of stay while maintaining the same safety, quality of aftercare, and low readmission rate achieved by standard postoperative hospitalization [5].

Healthcare systems operate increasingly under resource constraints, raising hospital costs even when length of stay is held constant. Successful ERAS programs are cost-effective and capable of reducing or limiting rising fees [6, 7]. Nonetheless, serious complications may occur following bariatric surgery; the 30-day major adverse event rate was 0–1.55%, as reported by systematic review [8]. The first priority in considering an ERAS program is that it does not exchange safety for efficiency or cost savings.

Fully ambulatory surgery, a potential next step for ERAS programs for a number of surgeries, has gained in popularity over the recent decade, particularly in France, where the national rate of ambulatory surgery across specializations increased from 36.2% in 2009 to 54.0% in 2016 [9]. Although bariatric surgery may be suited to an ambulatory approach, its rate of utilization remains low, < 5.0%. A possible option for moving toward safe, effective ambulatory bariatric surgery may be to extend a portion of the ERAS protocol into home care. The aim of this study, conducted in a French medical center, was to evaluate an ERAS protocol combined with a novel “Internet-connected surveillance” home follow-up to determine if it would result in the same level of safety as longer postoperative hospitalization.

## Methods

### Study Design

This study was a prospective observational study. At-home postoperative follow-up using a conventional questionnaire and a novel Internet application was employed to detect complications after early discharge following bariatric surgery.

### Patient Inclusion

Patients with morbid obesity > 18 years old and who met the accepted international criteria for bariatric surgery (i.e., US National Institutes of Health 1991 Guidelines [10]; European Guidelines [11]) underwent either one-anastomosis gastric bypass (OAGB) or sleeve gastrectomy (SG). All operations were performed after obtaining informed consent from each patient. The local institutional review board approved the present study.

### Perioperative Work-Up

Patients were assessed by a multidisciplinary surgical team involving surgeons, anesthesiologists, and nurses. The ERAS process was carefully explained to each patient. Our center’s detailed ERAS protocol has been previously described [12]. During preoperative consultation, patients were informed about the specific management strategy for follow-up, including early discharge at postoperative day one (with satisfactory postoperative progress) using Internet-connected at-home monitoring.

The day before surgery, patients were called by a medical secretary to confirm the hour of surgery. Patients were asked to perform “modern fasting,” which stipulates a light meal the night before surgery, and oral intake of a 400-mL carbohydrate-loaded drink 2 h before surgery.

### Intraoperative

No preoperative sedative was used, and anesthetic agents with a short duration of action were employed. Systemic intraoperative and postoperative analgesia was utilized to prevent postoperative pain, nausea, and vomiting, and to minimize narcotic use. Local anesthetics (e.g., ropivacaine 7.5 mg) were administered, and at port sites, intraperitoneal local anesthetic (IPLA) [13].

Compression socks were used intraoperatively rather than pneumatic stockings. Procedures were performed under laparoscopy by 2 experienced surgeons. Neither drains nor nasogastric tubes were used. Skin closure was performed with absorbable sutures and strips.

### Postoperative

After surgery, patients were supervised in the recovery room for about 1 h. Upon departure from the recovery room, the peripheral intravenous line was closed to facilitate patient movement. On return to the surgical unit, patients received no intravenous perfusion; only oral analgesia was administered. Patients were allowed to drink 4 h after surgery and were mobilized by the physiotherapist to walk.

On the first postoperative day, a blood test was performed to evaluate hemoglobin and C-reactive protein (CRP) levels. Prior to leaving the clinic, patients attended a nutritionist discharge meeting. Thrombophylaxis agents were selectively prescribed to high-risk morbidly obese patients (assessed postoperatively by Caprini score strictly > 3) [14]. Our postoperative thrombophylaxis protocol is reported in a previous study [15]. All patients were invited to a 1-month postoperative consultation where they were asked to complete a paper satisfaction survey.

### Technical Details

The connected surveillance is a “cloud-based” software as a service (SaaS) solution. It operates fully independently from any other information technology system. The hosting partner, certified by the French Ministry of Heath, has implemented an Information Security Management System (ISMS) for cloud operations, and follows the ISO27001:2013 standard. The platform comprises 2 modules as follows: a patient engagement module accessible to the patient as a native mobile application and a web application for all desktop and laptop computers and browsers. In addition, the patient’s care team also has access to a management application module to review the patient’s data and track his/her progress and health status.

The protection of personal data is ensured using multiple mechanisms. Access is restricted and protected with strong authentication. The platform uses role-based access control mechanisms to ensure staff access to personal data is only on a “need-to-know basis.” Data and communication are encrypted end to end, both in transit and at rest using advanced encryption algorithms (AES 256). Automatic monitoring systems are in place to identify external attempts to penetrate the security of the system. Electronic traffic check is permanently engaged. Data centers used are certified. Data are backed up in a separate geographical location to ensure that in the event of a significant problem in the main data center, service can be restarted from a secondary data center without significant loss of data.

### Study Period—At-Home Follow-Up

If patients met the discharge criteria, they were discharged from hospital the day after surgery. After discharge, patients participated in the 10-day follow-up study period (after which, they were given a classic bariatric surgical follow-up plan, including surgical consultations at 1, 3, and 6 months, and as needed). The 10-day study period was regulated by the Internet-connected surveillance protocol which was monitored 24 h per day by specially trained nurses. A total of 25 trained nurses were involved in the connected follow-up. The shift pattern used 2 teams and 2 12-h shifts to provide 24/7 coverage. The surveillance included data collection (patient-reported outcomes) and an automatic alert system to highlight abnormal events.

Patients were invited to choose a surveillance method to connect electronically to either the program website or mobile application. They were instructed to connect at least once a day to complete an online patient-oriented questionnaire (Table 3) reporting their current health status and likelihood of emerging complications. The solution promotes and tracks the patient’s ongoing adherence to follow-up. Patients received an alert at 9:00 am. They were required to complete the questionnaire before 1:00 pm. If a patient did not connect online, the application provided a reminder to the patient. If the patient did not complete the questionnaire at all, nurses received an alert as well and directly called the patient. The daily questionnaire aimed to capture any clinical side effects of surgery (e.g., fever, nausea, pain). The clinical results were sent to the surgeon daily by email; at any time, the surgeon could connect him/herself to the platform and review the details pertaining to any patient. Patients were also allowed to ask specific questions on the web chat or by telephone helpline. At all times, the patient was able to request a callback from nurses.

In order to assist the medical team in diagnosing patients based on their self-reported measures, objective values, such as serum level of CRP, were also systematically evaluated on day 1, 3, 5, and 7. In practice, patients left the hospital with blood test orders. The first blood test on postoperative day one was performed in the hospital. Subsequently, patients were asked to attend an ambulatory laboratory for blood work, and the patient reported the laboratory outcomes in the online platform. The surgeon defined the CRP cut-off level that designated an alert.

Any patient’s self-report or blood analysis that was out of the reference range for postoperative well-being was reported as an alert that the nurse and the surgeon received on their mobile phone through the connected surveillance system. They could elect to contact a patient for additional evaluation, as necessary. To avoid the system triggering a false positive alert, each participating surgeon configured his/her own protocol and adapted them in real time. Nonetheless, to limit costs due to unjustified alerts, each alert was first analyzed by the follow-up nurses who called the patient to clarify the alert.

### Statistical Analysis

Analyses were performed using the SPSS statistical package (version 20; IBM, Chicago, IL). Study analysis consisted of calculating simple counts and percentages for categorical data.

## Results

Between July 2016 and September 2017, from a total of 1083 bariatric surgery patients, 281 patients with morbid obesity who underwent RYGB or SG in our center entered the connected surveillance program. Among them, 17 patients had fewer than 10 days postoperative connected follow-up and therefore were excluded from the study. These 17 patients were not lost to follow-up, but attended their 1-month postoperative consultation and no complications were reported. A total of 264 completed the Internet-connected home monitoring. The mean age of patients was 40 ± 10.4 years (20–66) with a mean preoperative body mass index (BMI) of 42.4 ± 15.3 kg/m^2^ (36–54). Among the female-predominant cohort (236 females, 45 males), 126 (47.70%) underwent OAGB and 138 (52.3%) underwent SG. Mean length of hospital stay (LOS) for all operative procedures was 1.38 days (1–12). Specifically, 199/264 patients (75.4%) were discharged on postoperative day one; those hospitalized for ≥ 2 days were treated for minor complications. The primary surveillance tool chosen by 62.0% was the mobile phone application, while 38.0% chose the website for their communication with the nursing team.

### Complications

In the cohort of 264 who adhered to and completed the 10-day study, every complication sustained (100%) was detected by the connected surveillance protocol. Complications are presented in Table 2.

#### Unexpected Consultations

There were 22 (8.0%) unexpected complications (Table 2), defined as unscheduled surgery consultations prior the 1-month follow-up consultation. The great majority of patients (97.0%) who were readmitted (*n* = 30) were readmitted to the original hospital in which their surgery was performed. The consultations often led to CT scan and/or additional laboratory tests; yet, 0.0% of these consults required patient readmission. The cause of unexpected consults was abdominal pain and/or anxiety in 9 (41.0%), nausea in 6 (27.3%), wound infection/wound hematoma in 5 (23.0%), and colonic transit perturbation in 2 (9.0%). All of these complications were managed conservatively at home.

#### Readmissions

A total of 6 patients were readmitted for hospitalization. For 4 of them, no surgery-related complications were found after investigation (including upper endoscopy, repeat blood test, CT scan, and upper gastrointestinal X-ray).

The remaining 2 patients who were re-hospitalized had complications managed with non-surgical treatment. The first patient was asked to return to the emergency service because of intense epigastric pain. CT scan identified a portal thrombosis, and an urgent anticoagulation protocol was established during 5 days of hospitalization. The second patient proceeded to the nearest hospital because of abdominal pain associated with symptoms of shock. A subcapsular splenic hematoma was diagnosed and his surgeon was made aware of this hospitalization in another institution; an inter-hospital transfer was organized and the complication was managed conservatively. The patient completed the connected surveillance follow-up program.

#### Reoperations

Complications included 3 reoperations (1 staple line bleed, patient #1; 1 perigastric hematoma, patient #2; and 1 gastrocele, patient #3). Patient #1 presented with a low hemoglobin level on postoperative day one after SG. This patient underwent laparoscopy on day one, while he was still in hospital. The source of bleeding was the staple line, and cauterization of the bleeding point controlled the situation. Patient #2 developed a fever with diffuse abdominal pain on postoperative day 7 after SG. The laboratory finding showed a CRP of 153 mg/L. These clinical and laboratory findings triggered an alert and the patient was asked to return to emergency services. A CT scan identified a perigastric hematoma and laparoscopic drainage was performed. Patient #3 developed intense pain (pain scale 8/10) on postoperative day 3 after OAGB. A CT scan identified an image of a gastric mucocele. Urgent laparoscopy was performed for diagnosis and treatment. A part of the stomach had been excluded by mistake between two staple lines. This segment of stomach gradually distended due to persistent gastric secretion, and a mucocele developed. A total gastrectomy was performed.

### Satisfaction Survey

At 1 month following surgery, all the patients who completed the connected surveillance follow-up completed a satisfaction survey. Overall patient satisfaction was high: 96.0% reported that they were satisfied with this follow-up protocol (Table 1). The satisfaction survey revealed that patients appreciated the ease of use of the connected surveillance tools provided. Most importantly, patients reported feeling safe at home and were pleased that, in addition to the mobile or website surveillance, the call helpline was always available to them and provided a sense of security throughout the 10-day program (Tables 2 and 3).

**Table 1:** Connected surveillance satisfaction survey outcomes

Patient study follow-up statements	Patients who agreed with statements n* (%)
Appreciated connected surveillance ease of use	232 (88.0)
Felt safe at home	241 (91.0)
Called helpline at least once	171 (65.0)
Satisfied with connected surveillance follow-up	254 (96.0)

^*^Based on the patients (*n* = 264) who completed the 10-day study

**Table 2:** Most common causes of consultation

Main symptom	Emergency consultation *n* (%) (*n* = 22)	Hospitalization for investigation *n* (%) (*n* = 6)	Operative management *n* (%) (*n* = 3)
Abdominal pain	9 (41)	3 (50)	3 (100)
Nausea/vomiting	6 (27)	2 (33)	0
Wound complication	5 (23)	0	0
Transit problems	2 (9)	0	0
Hematemesis	0	1 (17)	0

**Table 3:** Post-surgical discharge Internet-connected surveillance questionnaire

Section questionnaire	Daily question	Identified as an alert
Pain	Do you have pain? Please use the “pain scale measure” to tell us how painful it is.	> 6/10
Abdominal discomfort	Do you have nausea or vomiting? How many times a day?	> 3 times a day
Clinical sign	Do you have fever? How high is the fever	> 38 °C
Hydration level	How much do you drink a day?	< 1000 cc
Biological sign	Enter your CRP level	> 120 or abnormal kinetic pattern

## Discussion

### Closely Monitored Aftercare Is Critical

Roux-en-Y gastric bypass (RYGB) and SG are the most frequently used bariatric procedures in France, with the greatest number of bariatric operations performed (12.0%) in a small number of centers [16]. Pre- and postoperative care regimens are well standardized and the inpatient mortality rate is low (< 0.2%). Bariatric management of obesity is, thus, generally considered to be quite safe [17]. However, as identified by systematic review [8], serious complications (e.g., bleeding, leak) in up to 1.55% of cases can occur following bariatric surgery. Early hemorrhage (< 48 h postoperative) has been reported with a rate 0–5.0% [18], and early leak (< 30 days) has been found in 0.84–1.43% of cases following RYGB, and in 0.23–2.19% of cases after SG [8]. As the number of bariatric procedures increases worldwide, we can expect to manage a higher number of patients with complications.

### Early Complication Detection

Particularly when using an ERAS discharge protocol, early diagnosis of any complication after bariatric surgery is essential. Achieving close monitoring of patients after early discharge is a challenge. Patients with suspected complications must be referred for a medical consultation as soon as possible. In the current study, the primary outcome of interest—detection of early complication—was identified in 100% of cases by the “connected surveillance” follow-up.

When a bariatric patient experiences an early complication, he/she must be able to be in touch rapidly with his/her original bariatric program. The patient’s bariatric program is extensively familiar with the diagnosis and urgent management of complications that may emerge, and also with each individual patient’s pre- and intraoperative history. Nonetheless, bariatric surgical patients often present to the nearest hospital irrespective of whether it has a bariatric surgeon on staff. Almost all patients in our study were readmitted to their original center as a direct result of their connected surveillance instruction and continual daily communication with the center.

There were 22 consultations, with an overall study readmission rate of 2.0% (6/281). This rate was higher than those observed in the literature [19, 20]; yet, the published rate may be underestimated due to the loss of follow-up in other studies that is associated with readmission to hospitals that are not the primary bariatric program. To reduce this challenge, the current surveillance program emphasized acquisition of objective clinical values (e.g., serum CRP and white blood count levels) at day 1, 3, and 5 throughout the 10-day follow-up to alert the surgeon of emerging postoperative complications. CRP assessment can detect leak and abscess with a high level of sensitivity and specificity [21].

Instituting an ERAS program requires a commitment to multidisciplinary team involvement [22]. Utilizing a connected surveillance post-discharge protocol with a specialized nursing and surgeon team may provide the patient support required for proper management of emerging complications. In addition, this study showed that successful early discharge with very close postoperative follow-up appeared to be a cost-effective solution. Indeed, the average cost of this 10-day-connected surveillance program is approximately 10 times lower than to extend in-hospital stays beyond the first 24 h. By improving patient safety and reducing rising fees, the study results convinced our hospital to adopt connected surveillance to enhance the ERAS program as a new standard of care.

### Limitations

A limitation of this study was that it was not a randomized controlled trial, which prevented controlling for potentially confounding variations in patient characteristics and comparing outcomes of those who underwent connected surveillance protocol with a control group of patients.

## Conclusion

In a bariatric surgery program using an Enhanced Recovery after Surgery (ERAS) protocol, “Internet-connected surveillance” proved to be safe and effective in detecting all (100%) early complications, and patients were very satisfied with their care.
